# Genotype–phenotype correlations in 18 European patients with heterozygous *KIF1A* variants: key considerations for assessing *KIF1A* variant causality

**DOI:** 10.3389/fmed.2025.1704209

**Published:** 2026-01-08

**Authors:** Anna Uhrova Meszarosova, Elea Galiart, Petra Lassuthova, Konstantinos Kolokotronis, Benjamin Seidl, Alena Musilova, Anna Peckova, Alena Takacsova, Emilie Vyhnalkova, Dagmar Grecmalova, Eva Vlckova, Vladana Skutilova, Katharina Steindl, Anita Rauch, Georg M. Stettner, Dana Safka Brozkova

**Affiliations:** 1 Neurogenetic Laboratory, Department of Paediatric Neurology, Second Faculty of Medicine Charles University and University Hospital Motol, Prague, Czechia; 2 Neuromuscular Center Zurich and Department of Paediatric Neurology, University Children’s Hospital Zurich, University of Zurich, Zurich, Switzerland; 3 Institute of Medical Genetics, University of Zurich, Schlieren, Switzerland; 4 Department of Medical Genetics, Masaryk Hospital, Ústí nad Labem, Czechia; 5 Department of Medical Genetics and Genomics, Faculty Hospital, Brno, Czechia; 6 Department of Biology and Medical Genetics, Second Faculty of Medicine Charles University and University Hospital Motol, Prague, Czechia; 7 Institute of Molecular and Clinical Pathology and Medical Genetics, University Hospital Ostrava and Institute of Laboratory Medicine, Medical Faculty, Ostrava, Czechia; 8 Department of Neurology, Masaryk University and University Hospital Brno, Brno, Czechia; 9 Department of Molecular Biology, Faculty Hospital Hradec Kralove, Hradec Kralove, Czechia

**Keywords:** *KIF1A*-associated neurological disorder, spastic paraplegia, neuropathy, neurodegeneration, *KIF1A* pathogenic variants

## Abstract

**Background:**

Variants in the *KIF1A* have been associated with a wide range of phenotypes. Most variants are found in the protein’s motor domain. The clinical phenotype of *KIF1A*-associated disorders (KAND) correlates with the position and the type of variant. Missense variants in the motor domain are predominantly associated with severe phenotypes and often occur *de novo*.

**Methods:**

Patients from the Czech Republic and Switzerland were identified during DNA diagnostics for neuromuscular or neurodevelopmental disorders. Clinical and genetic data were analyzed retrospectively.

**Results:**

A total of 18 patients with heterozygous *KIF1A* variants were reported. The clinical spectrum ranges from a very severe congenital phenotype to mild spastic paraplegia or early-onset slowly progressive neuropathy. Among patients with early clinical manifestation (n = 13; congenital symptoms, gross motor delay, complicated/pure spastic paraplegia, neuropathy), all detected variants were missense and localized in the motor domain, eight times confirmed to be *de novo*. In individuals with adult onset (*n* = 5; all spastic paraplegia), a frameshift variant outside the motor domain was also detected in one case.

**Conclusion:**

KAND phenotypes are not only limited to severe and early-onset phenotypes but also include adult-onset less severe ones. The localization of a missense *KIF1A* variant in the motor domain corresponds with a more severe disease, but not exclusively. Given the broad phenotypic spectrum associated with *KIF1A* variants, each variant should be individually evaluated for pathogenicity. Based on our findings, we propose a supporting algorithm outlining key considerations to support variant causality and the prediction of the associated phenotype.

## 1 Introduction

The kinesin family member 1A gene (*KIF1A*) encodes the microtubule-associated motor protein KIF1A, which belongs to the kinesin-3 subfamily. Kinesins play essential roles in neuronal function by transporting cargoes in the anterograde direction along microtubules (1). KIF1A is abundantly expressed in the axons of the neurons in the brain and spinal cord.^1^ The phenotype of *KIF1A* knockout mice exhibits motor and sensory impairments, reduced synaptic vesicle density in nerve terminals, and the accumulation of clear vesicles in nerve cell bodies (2). In humans, pathogenic *KIF1A* variants have been linked to a broad spectrum of neurological disorders, which are summarized under the umbrella term of KIF1A-associated neurological disorders (KANDs) (3). Initially, biallelic *KIF1A* variants were described with the phenotype of hereditary spastic paraplegia (SPG30) and hereditary sensory and autonomic neuropathy (HSAN2C) (4–6). Later, inherited or *de novo* monoallelic (heterozygous) *KIF1A* variants were associated with pure (uncomplicated) or complicated hereditary spastic paraplegia (7–9) and finally *de novo KIF1A* variants with severe neurodegenerative conditions or a broader range of clinical phenotypes grouped under the term NESCAV syndrome (neurodegeneration and spasticity with or without cerebellar atrophy or cortical visual impairment; formerly also classified as autosomal dominant intellectual disability type 9 [MRD9]) and/or PEHO syndrome (progressive encephalopathy with oedema, hypsarrhythmia, and optic atrophy) (10–13). Recently, hypotonia, spasticity, ataxia, epilepsy, optic nerve and cerebellar atrophy, and cognitive impairment have been reported as the most common clinical features in KAND patients (14). The KAND spectrum shows significant differences in terms of the severity of clinical symptoms and age of onset (from congenital or infantile onset to adult onset). In general, congenital/early-onset monoallelic cases exhibit a faster degenerative and severe disease progression than adult-onset and biallelic cases (15, 16).

KIF1A consists of several protein domains, of which the motor domain is of particular importance for the pathogenesis of KAND. The kinesin motor domain is responsible for transport processes along the axon, and the severity of clinical symptoms appears to depend on the presence of a variant in this particular protein domain (17). Pathogenic variants localized in the motor domain have more disruptive effects on the proper function of the protein when compared to variants in other protein domains (18–20). The vast majority of described pathogenic variants are localized in the motor domain and often occur *de novo* (3, 17). A total of 129 pathogenic *KIF1A* variants (mostly missense variants, only 20 truncating) have been reported, mainly resulting in spastic paraplegia (pure or complicated), several variants with severe complex phenotype, and a few variants with mild neuropathy (according to HGMD Profess. 2025.2 version). Missense variants are thought to have a dominant-negative effect, in which a slight conformational change results in non-functional protein that replaces the normal, functional protein (21). Consequently, missense *KIF1A* variants may have more severe phenotypic effects than truncating variants (22). This observation contrasts with the ACMG/AMP classification guidelines and standard variant-pathogenicity predictions based on amino acid and protein conformation changes, which may not be relevant in the case of the *KIF1A* gene.

In this study, we report on 18 patients from 15 families of European descent with a heterozygous *KIF1A* variant; eight were confirmed as *de novo* and include five novel variants. The clinical spectrum ranges from severe phenotypes (including congenital) with spasticity, hypotonia, ataxia, epilepsy, and cognitive decline to pure spastic paraplegia or neuropathy with varying degrees of disability. Based on our results, we propose a supportive algorithm for the assessment of pathogenicity/causality of *KIF1A* variants with the expected phenotype.

## 2 Patients and methods

### 2.1 Patients

Participants with *KIF1A* variants in this retrospective cohort study were recruited in two centers:

- Neurogenetic laboratory, Department of Paediatric Neurology of the Second Faculty of Medicine, Charles University and Motol University Hospital, Prague, Czech Republic. The Neurogenetic Laboratory is the core facility in the Czech Republic providing comprehensive genetic testing of neuromuscular disorders connected to the ERN-RDN European Reference Network; and
- Paediatric Neuromuscular Center Zurich, Department of Paediatric Neurology, University Children’s Hospital Zurich, University of Zurich, Zurich, Switzerland. The Neuromuscular Centre Zurich is a national reference center for rare neuromuscular diseases, accredited by Kosek (Nationale Koordination Seltene Krankheiten / National Coordination of Rare Diseases).

### 2.2 Genetic testing

For the patients examined in the Czech Republic, the coding regions, as well as the exon–intron boundaries up to 10 base pairs into the intron of all genes associated with (i) hereditary spastic paraplegias and neuropathies or (ii) all OMIM genes, were analyzed. NGS libraries were prepared with the SureSelect Exome v.5, v.6, or v.8 kit (Agilent Technologies, CA, United States) or Twist Exome 2.0 plus Comprehensive Exome Spike-In (TWIST Bioscience, CA, United States). FASTQ data were analyzed twice independently using (i) an in-house pipeline based on the Genome Analysis Toolkit (GATK) best-practices protocol (23) with Annovar software annotations^2^ and (ii) SureCall software (Agilent Technologies, CA, United States) with QIAGEN Ingenuity Variant analysis software^3^ evaluation. For patients examined in Switzerland, the coding regions of all genes associated with neurodevelopmental disorders and the exon-intron boundaries up to 20 base pairs into the intron, were analyzed using DRAGEN V4.0 (Illumina, CA, United States) and NxClinical 6.2 (BioDiscovery, Inc.). Sanger sequencing was used for segregation analysis. Variants were named according to the transcript NM_001244008.2.

The following aspects were considered to assess causality: *de novo* occurrence (or segregation analysis in family where available), phenotype correlation with previously reported according to HGMD Profess.^4^ Frequency (allele count) in GnomAD version v4.1 (24) reported in ClinVar,^5^ and several prediction tools (ACMG classification by GeneBe, AlphaMissense) (25–27). Six polymorphic microsatellite markers (in-house designed) were used for parentage testing for *de novo* occurrence.

### 2.3 Phenotype description

The source for data extraction was the clinical information system of the two centers. The following data were extracted from the clinical information system: *KIF1A* variant and characteristics (localization within the protein, results from segregation analysis if available), main clinical phenotype, sex, age, and symptoms at onset of the disease, age at first examination, presence of specific symptoms (spasticity, peripheral neuropathy, walking impairment, ataxia, upper limb impairment, dysarthria, global developmental delay and/or intellectual disability, cognitive decline), imaging results (brain and/or spinal cord MRI), and other relevant features (e.g., epilepsy).

Written informed consent was obtained from all patients, or their legal guardians, prior to their inclusion in the study and prior to disease-related genetic testing. This study meets the criteria and principles of the Declaration of Helsinki, and ethical board approval was obtained from all participating facilities.

## 3 Results

### 3.1 Clinical findings

We report 18 patients (7 males, 11 females) from 15 families with a heterozygous *KIF1A* variant. In patients #17 and #18 (Table 1), the pathogenicity of the identified variants could not be sufficiently confirmed. Therefore, their clinical and genetic data are reported; however, they were excluded from the subsequent summary dataset presented in Table 2. Both individuals presented with late-onset disease and a phenotype consistent with pure spastic paraparesis. Among the remaining 16 KAND patients (#1–#16; Table 1), the age of onset demonstrated a bimodal distribution in: (i) early childhood (12/16) or even congenitally (1/16) or (ii) adulthood (3/16; 20–42 years). The main clinical diagnosis was pure or complicated spastic paraplegia with varying clinical signs and severities, including upper limb impairment, ataxia, epilepsy, intellectual disability, and cognitive decline. Two patients (#14 and #16) presented with very severe phenotypes; patient #14, who had even congenital symptoms and a complex phenotype of severe congenital axonal neuropathy without spasticity, died of respiratory failure at 3 months of age. The main clinical diagnosis of patient #12 was ataxia with delayed gross motor development. Patient #4 was diagnosed with axonal neuropathy confirmed by electromyography (EMG). Her initial symptoms appeared in early childhood as frequent tripping. At 39 years of age, she is able to walk without support, and the disease has followed a slowly progressive course (See Table 1 for details).

**Table 1:** Summary of *KIF1A* variants and phenotypes in 18 KAND patients.

Pathogenicity of KIF1A variant	Pathogenic																Uncertain	
Patient nr.	1 (sister of Pt. 2, mother of Pt. 3)	2 (sister of Pt. 1)	3 (son of Pt.1)	4	5 (son of Pt. 6)	6 (mother of Pt. 5)	7	8	9	10	11	12	13	14	15	16	17	18
Main clinical diagnosis	Pure SPG	Pure SPG	Pure SPG	Axonal neuropathy	Pure SPG	Pure SPG	Complex SPG	Complex SPG	Pure SPG	Complex SPG	Complex SPG	Ataxia	Complex SPG	Severe congenital HMSN, respiratory failure, died at 3 months	Complex SPG	Complex SPG	Pure SPG	Pure SPG
Variant [heterozygous]	c.110T>C	c.110T>C	c.110T>C	c.232G>A	c.499C>T	c.499C>T	c.647G>A	c.761G>A	c.761G>A	c.773C>T	c.821C>T	c.914C>T	c.914C>T	c.920G>A	c.939G>C	c.946C>T	c.925 T>A	c.871G>A c.3363_3364del*
KIF1A variant location	Motor domain	Motor domain	Motor domain	Motor domain	Motor domain	Motor domain	Motor domain	Motor domain	Motor domain	Motor domain	Motor domain	Motor domain	Motor domain	Motor domain	Motor domain	Motor domain	Motor domain	Motor domain [only c.871G>A]
Segregation analysis/familial occurrence	Familial (affected sisters and father)			Healthy mother and brother, wild type, healthy father not tested	Familial (affected mother and son)		Unknown	*De novo*	Familial (both parents with a diagnosis of early-onset spastic paraparesis /cerebral palsy; variant inherited from father†)	*De novo*	*De novo*	*De novo*	*De novo*	*De novo*	*De novo*	*De novo*	unknown	unknown
Sex	F	F	M	F	M	F	M	F	F	M	F	M	F	F	M	F	M	F
Age at onset [years]	42	35	20	<5	4–5	4	<1	<1	<1	<1	<1	<1	<1	Antenatal	6–8 (early school age)	<1	30+	50+
Age at first examination [years]	?	?	22	39	4–5	9	<1	<1	9	2	<1	<1	<1	0	10	<1	53	52
Symptoms at onset	Spastic gait with fallings	Abnormal gait	Spastic paraparesis	Tripping	Abnormal gait	Abnormal gait	Muscular hypotonia, gross motor developmental delay, ataxia	Spastic paraparesis, epilepsy	Spastic paraparesis	Gross motor developmental delay	Abnormal gait	Gross motor developmental delay		Poor neonatal adaptation, continual respiratory support (CPAP) required, severe distal flaccid paresis	Abnormal gait	Severe global developmental delay, spasticity, epilepsy	gait difficulties	spastic paraparesis
Spasticity	+	+	+	–	+	+	+	+	+	+	+	+	+	−	+	+	+	+
Peripheral neuropathy	Mild	?	–	+	–	–	–	+	–	–	−	Unknown		+ (axonal)	+ (axonal)	Unknown	–	−
Gait impairment	Severe, uses wheelchair	Severe, uses wheelchair	Spastic gait, walk without support	Walk without support	Mild, walk without aids	Mild, walk without aids	+	Severe, uses crutches	Severe, uses crutches	+	Severe, walk with support	Walk with support	Spastic / atactic gait	n.a.	Severe, uses wheelchair	Walk with support	mild, walk without aids	mild
Ataxia	mild	−	−		−	−	+	−	−	−	−	+	+	n.a.	−	+	−	−
Upper limb impairment	−	−	−	Mild muscle atrophies	−	−	Dysmetria	−	−	Spasticity	−	Dysmetria	–	Severe distal flaccid paresis	Fine motor impairment	Tremor, dysmetria, spasticity	spasticity, hyperreflexia	−
Dysarthria	–	−	−	−	−	−	–	?	−	–	–	–	–	n.a.	+	−	−	−
Incontinence	+	–	−	−	?	?	+	−	?	−	?	+		n.a.	+	+	−	?
Global developmental delay / Intellectual disability	−	–	−	−	–	–	+	Mild intellectual disability	Mild intellectual disability	Motor developmental delay / IQ 76–86 borderline	Intellectual disability	+ / borderline (developmental quotient 70)	+	n.a.	Intellectual disability	+	−	−
Cognitive decline	–	−	−	−	Mild decline	−	−	−	−	−	−	−		n.a.	Severe decline	−	−	−
MRI brain/spine	Not performed	Normal	Not performed	Not performed	Not performed	Not performed	Cerebellar atrophy	Not performed	Not performed	Brain: hypoplasia CC, accentuated cerebellar fissure, spine: normal	Normal	Accentuated cerebellar fissure	Cerebellar hypoplasia	Not performed, brain ultrasound without pathological finding	Normal	Progressive cerebellar atrophy	brain: deposits of paramagnetic substances in the substantia nigra bilat, crus cerebri and right basal ganglia, spine: normal	mild thoracic spine atrophy
Other	−	Optical nerves atrophy	−	−	–	–	Visual impairment, challenging behavior	–	–	–	–	Limited vocabulary	Optic nerve hypoplasia, strabismus	–	Aggressiveness	Limited vocabulary, growth hormone deficiency	–	–

*Compound heterozygous, †No detailed clinical information available. SPG spastic paraparesis/paraplegia, n.a., not analysed/applicable, Pt. Patient.

**Table 2:** Summary of symptoms of reported KAND patients.

KAND patients		A	B	
		All *n* = 16 [100%]	Early/congenital onset *n* = 13 [100%]	Adult onset *n* = 3 [100%]
Age at onset	Congenital or early childhood	13 [81%]	n.a.	n.a.
	Adulthood	3 [19%]	n.a.	n.a.
Walking	Unable/with aids	10 [63%]	8 [62%]	2 [67%]
	Able without aids/no limitation	6 [37%]	5 [38%]	1 [33%]
Spasticity		14 [88%]	11 [85%]	3 [100%]
Neuropathy (only)		2 [13%]	2 [15%]	–
Spasticity + neuropathy		3 [19%]	2 [15%]	1 [33%]
Ataxia		5 [31%]	4 [31%]	–
Dysarthria		1 [6%]	1 [8%]	–
Upper limb impairment		7 [44%]	7 [54%]	–
Incontinence		5 [31%]	4 [31%]	1 [33%]
Epilepsy		2 [13%]	2 [15%]	–
Global developmental delay/intellectual disability		10 [63%]	10 [77%]	–
Cognitive decline		2 [13%]	2 [26%]	–

The data from patients #17 and #18 were not included as the pathogenicity of the *KIF1A* variant was not fully confirmed: (A) all patients and (B) divided according to age at onset. n.a., not applicable.

Spasticity was present in 88% of patients (14/16), and in 3 of them, it was combined with neuropathy. In the remaining two patients, the primary manifestation was axonal neuropathy. The degree of walking impairment varied from an inability to walk to a mild impairment with minor limitations. Of all the reported patients, 63% (10/16) were unable to walk (wheelchair dependent) or required assistive devices. The degree of gait impairment in the reported cohort does not depend on whether the disease had an early or adult onset; in both groups, two-thirds of patients were either unable to walk or required assistive devices, while one-third walk unaided. Global developmental delay and/or intellectual disability was observed only in patients with early onset, and it accounted for 77% (10/13).

In terms of other clinical symptoms, 44% (7/16) of patients had upper limb impairment. Incontinence was reported in 31% (5/16). Two patients suffered from epilepsy. Dysarthria was noted only in one patient, and in three patients, visual impairment or optic nerve atrophy/hypoplasia with strabismus was present. Two patients exhibited challenging behavior or aggressiveness (see Tables 1, 2, for details).

MRI examinations of the brain or spinal cord were performed on nine patients. In five patients, changes/atrophies of varying degrees were revealed and accounted for 56% (5/9). In five early-onset cases, the cerebellum was affected, primarily showing hypoplasia or atrophy. Clinical symptoms are summarized and described in detail in Tables 1, 2.

### 3.2 Genetic findings

A total of 14 different heterozygous *KIF1A* variants were detected. Eleven variants are assumed to be pathogenic and causal (Tables 1, 3). Eight variants were confirmed with the *de novo* occurrence. Two variants—c.761G>A and c.914C>T—were each detected twice across four index patients (mostly *de novo*). Two variants—c.821C>T and c.939G>C—were not previously documented; both were detected in two index patients with early-onset and relatively severe phenotypes. Variant c.110T>C segregated with the disorder in three family members (#1, #2, #3) with adult-onset pure spastic paraplegia. Variant c.499C>T was detected in patient #5 and his mother #6 (both affected from childhood), as well as in a healthy sister aged 20 years. Although segregation of the variant is inconsistent, the variant is considered causative (discussed in detail later).

**Table 3:** Detected *KIF1A* variants, characteristics, and predictions.

Variant’s pathogenicity	Patient nr.	Heterozygous variant NM_001244008.2	Protein change	Segregation analysis/familial occurrence	Protein domain presence	HGMD accession/variant class	GnomAD_total (v4.1) /alelle frequency/	ClinVar (ID)	GeneBeprediction	ACMGgenerated by GeneBe	Alpha missensescore	Alpha missenseprediction	HGMD; diagnosis described	Assumed causality
Pathogenic	1, 2, 3	c.110T>C	p.(Ile37Thr)	Familial/present in affected sister, affected father not tested	Kinesin motor	CM1810577/DM	–	Uncertain significance (871018)	Uncertain significance	PM2, PP3	0.98	Likely_pathogenic	Described 3x, [1x *de novo*]; spastic paraplegia	Pathogenic
	4	c.232G>A	p.(Gly78Ser)	Healthy mother and brother wild type, healthy father not tested	Kinesin motor	CM200409/DM	0.00012%	Pathogenic/likely pathogenic (373905)	Pathogenic	PM1, PM2, PM5, PP3_Moderate, PP5_Very_Strong	0.84	Likely_pathogenic	Described 2x [1x *de novo*]; spastic paraplegia	Pathogenic
	5, 6	c.499C>T	p.(Arg167Cys)	Familial / affected mother, son, healthy daugter (age 20) all heterozygous	Kinesin motor	CM150181/DM	0.000657%	Pathogenic/likely pathogenic (162055)	Pathogenic	PM1, PM2, PM5, PP3_Strong, PP5_Very_Strong	0.93	Likely_pathogenic	Described 4x, [1x*de novo*]; spastic paraplegia	Pathogenic
	7	c.647G>A	p.(Arg216His)	Unknown/n.p.	Kinesin motor	CM157165/DM	–	Pathogenic/likely pathogenic (208161)	Pathogenic	PM1, PM2, PM5, PP3_Strong, PP5_Very_Strong	0.99	Likely_pathogenic	Described 12x [8x *de novo*]; developmental disorder/delay/cerebellar ataxia/spastic paraplegia/neurodegeneration and spasticity	Pathogenic
	8, 9	c.761G>A	p.(Arg254Gln)	Patient 8: *de novo* Patient 9: familial/uninformative, both parents under diagnosis spastic paraparesis/cerebral palsy, variant inherited from father	Kinesin motor	CM1514258/DM	–	Pathogenic/likely pathogenic (162055)	Pathogenic	PM1, PM2, PM5, PP3_Strong, PP5_Very_Strong	0.99	Likely_pathogenic	Described 11x [5x *de novo*]; childhood cerebellar hypoplasia and atrophy/Neurodevelopmental disorder/spastic paraplegia/NESCAV syndrome	Pathogenic
	10	c.773C>T	p.(Thr258Met)	*De novo*	Kinesin motor	CM1714233/DM	–	Pathogenic/likely pathogenic (464261)	Pathogenic	PM1, PM2, PM5, PP3_Moderate, PP5_Very_Strong	0.98	Likely_pathogenic	Described 6x [1x*de novo*]; neurological disorder/neuropathy/dyskinesia/dystonia	Pathogenic
	11	c.821C>T	p.(Ser274Leu)	*De novo*	Kinesin motor	Novel	–	Pathogenic/likely pathogenic (211298)	Pathogenic	PM1, PM2, PP3_Moderate, PP5_Very_Strong	1.00	Likely_pathogenic		Pathogenic
	12, 13	c.914C>T	p.(Pro305Leu)	*De novo*	Kinesin motor	CM1921975/DM	–	Pathogenic/likely pathogenic (428604)	Pathogenic	PM1, PM2, PP3_Moderate, PP5_Very_Strong	0.93	Likely_pathogenic	Described 13x [6x *de novo*]; developmental disorder/congenital ataxia/dystonia	Pathogenic
	14	c.920G>A	p.(Arg307Gln)	*De novo*	Kinesin motor	CM1514260/DM	–	Pathogenic/likely pathogenic (418275)	Pathogenic	PM1, PM2, PM5, PP3_Strong, PP5_Very_Strong	0.95	Likely_pathogenic	Described 9x [5x *de novo*]; spastic paraplegia with CNS involvement/cerebellar hypoplasia/spastic paraplegia	Pathogenic
	15	c.939G>C	p.(Trp313Cys)	*De novo*	Kinesin motor	Novel	–	–	Likely pathogenic	PM1, PM2, PP3_Strong	1.00	Likely_pathogenic		Pathogenic
	16	c.946C>T	p.(Arg316Trp)	*De novo*	Kinesin motor	CM150188/DM	–	Pathogenic/likely pathogenic (162060)	Pathogenic	PM1, PM2, PM5, PP3_Strong, PP5_Very_Strong	0.99	Likely_pathogenic	Described 13x [5x *de novo*], severe impairment/spastic paraplegia/NESCAV syndrome	Pathogenic
Uncertain	17	c.925T>A	p.(Ser309Thr)	Unknown/not present in healthy brother	Kinesin motor	Novel	–	–	Pathogenic	PM1, PM2, PM5, PP3_Strong	0.76	Likely_pathogenic		Uncertain (insufficient segregation)
	18*	c.3363_3364del	p.(Leu1222Aspfs*22)	Unknown/n.p.		Novel	–	–	Pathogenic	PVS1, PM2	–	–		Uncertain (missing segregation)
Likely benign		c.871G>A	p.(Gly291Arg)	Unknown/n.p.	Kinesin motor	–	0.00007142%	Uncertain significance (1307488)	Uncertain significance	PM2	0.50	Ambiguous		Likely benign (frequency, predictions)

*Compound heterozygous; n.p. not performed.

The causality of variants c.925T>A and c.3363_3364del was uncertain because it was not possible to perform a segregation analysis in patients with adult onset, as family members were not available or deceased. Variant c.871G>A is assumed to be rather benign because of its frequency and benign predictions (Table 3).

The genetic aspects of the detected *KIF1A* variants are summarized in detail in Table 3. All variants except one were missense and were localized in the kinesin motor domain, and all patients were heterozygotes for the *KIF1A* variant. The only exception is patient #18, who had adult-onset pure spastic paraplegia and was compound heterozygous for one missense variant, c.871G>A, and one truncating variant, c.3363_3364del, localized outside the kinesin motor domain. Only the truncating variant, c.3363_3364del, was considered pathogenic [Pedigrees of all reported patients in Supplementary material 1].

## 4 Discussion

### 4.1 Genotype–phenotype correlation

We summarized the clinical and genetic aspects of 18 patients with *KIF1A* variants and assessed the causality of each variant. We described that *KIF1A* variants can be associated with a very severe and early lethal course (e.g., patient #14 harboring a *de novo KIF1A* variant). In the reported cohort, spasticity and complex spastic paraplegia were the most common phenotypes. The majority of the described patients manifested a more severe phenotype with early onset. However, even in early-onset cases, a milder phenotype with slow progression may be observed (as in patient #4). All patients carry a variant within the motor domain, except for one truncating variant located outside this domain, which is consistent with previous reports (15, 17).

A *KIF1A* variant confirmed to be *de novo* in a patient with a severe early-onset phenotype can be considered causal and pathogenic. Moreover, if the variant has been previously described in association with a KAND, it provides additional support for pathogenicity. Nevertheless, determining variant causality can be challenging even in early-onset cases. The family with patients #5 and #6 (heterozygous for the c.499C>T variant) illustrates this complexity. The symptomatic patient #5 and his mother #6 have suffered from spasticity and an abnormal gait since childhood. However, the sister of patient #5, who is also heterozygous for this variant, is free of symptoms at the age of 20. This variant has been described two times in the literature, both *de novo* and with autosomal dominant inheritance, and with variable age of symptom onset (13) (HGMD Access. CM150181) and with autosomal dominant inheritance. Rudenskaya et al. (28) reported recently that the variant segregated in five affected family members who manifested the disorder at very different ages. In addition, Gallagher et al. described this variant in a 50-year-old patient in whom the gait impairment began in childhood and progressed slowly (29).

Determining the causality of *KIF1A* variants can be challenging in patients with adult-onset, slow progression, or neuropathy. These features are not typically associated with KAND, although they were reported previously (16, 30, 31). Moreover, the segregation analysis is often incomplete or unavailable for adults. Therefore, pathogenicity remains uncertain in patients #17 and #18.

Patients #1, #2, and #3 (son, mother, and maternal aunt) exhibited adult-onset spastic paraplegia (the son was in early adulthood ~20 years). The c.110T>C variant has been previously reported in an autosomal dominant SPG pedigree (32) and also *de novo* in a sporadic case (33); however, age at onset was not specified in either study.

Patient #4, who presented with axonal neuropathy (sporadic patient in the family), experienced the first symptoms in childhood (frequent tripping), with slow disease progression and a mild course. Neuropathies have been reported only rarely as a KAND phenotype (32, 34–36). We consider the variant c.232G>A in patient #4 to be causal because it has been reported as pathogenic, including *de novo* occurrence (9, 37), although the segregation analysis in the family is incomplete.

Interestingly, all patients in our cohort with a mild disease course (#1–#6) harbor variants located at the beginning of the motor domain (in our case, up to nucleotide c.499). Boyle et al. have previously reported that the specific localization within the motor domain may influence the severity of clinical manifestations (3). Rao et al. (20) showed that different amino acid substitutions are associated with different biophysical and clinical outcomes. Both the structural change and the properties of the substituted amino acid determine the extent of KIF1A motor dysfunction. These mutation-specific differences in KIF1A motility correlate with neurodevelopmental severity.

Truncating *KIF1A* variants have rarely been described as a cause of KAND. Only 14 truncating pathogenic *KIF1A* variants have been described [according to HGMD Profess., 07/2025]. They are more frequently localized outside the motor domain and are hypothesized as leading to haploinsufficiency or decreased final protein levels with a loss-of-function effect (opposite to the missense variant with dominant negative effect) (9, 19, 20, 31).

Patient #18 carries a truncating *KIF1A* variant c.3363_3364del together with a missense variant c.871G>A. We assume that only the truncating variant is pathogenic because the c.871G>A variant is present in the GnomAD v4.1 population database with a low frequency of 0.0007142% and has benign predictions. Nevertheless, an autosomal recessive mode of inheritance could also be considered (although the cis/trans position was not confirmed, since family members were not available for segregation analysis). An autosomal recessive inheritance has been reported rarely with hereditary sensoric neuropathy caused by *KIF1A* variants and only for truncating variants; all these variants were localized out of the kinesin motor domain (14, 16, 38).

### 4.2 Key considerations for assessing *KIF1A* variant causality

Assessing the causality of *KIF1A* variants is complex but essential for accurate genetic counselling and effective disease management. In our cohort, the pathogenicity of certain variants (e.g., c.499C>T, due to a healthy heterozygous carrier; c.110T>C, due to late onset and long-time lasting (years) segregation) remained uncertain for some time and was initially classified as unresolved. Conversely, several variants that initially appeared likely pathogenic were ultimately excluded based on one or several of these factors: lack of segregation, non-motor domain localization, benign predictions, or rather higher population frequency (unpublished data).

Based on the literature data, observed genotype–phenotype correlations, and our clinical experience, we propose a supportive algorithm with key considerations to aid in *KIF1A* variant classification and phenotype prediction. Although it overlaps partially with ACMG/AMP guidelines, it integrates gene-specific features and is intended as a practical, not definitive, interpretive tool (Table 4).

**Table 4:** Supportive algorithm with key considerations for assessing the causality of *KIF1A* variants (both AD and AR inheritance were considered, but missense variants were described only with AD).

Variant	Missense						Truncating			
Presence in the motor domain	Yes		No		Yes	No	Yes	No	Yes	No
Frequency/allele count in GnomAD v4.1 total	0–5 alleles				> 5 alleles		0–5 alleles		> 5 alleles	
Segregates with disease/*de novo*	Yes	No	Yes	No	No (very likely)		Yes	No	No (very likely)	
Expected causality	Likely pathogenic	Uncertain compared with reported cases, age of onset can intrafamilially differ!	Likely pathogenic	Likely benign	Likely benign		Likely pathogenic	Likely benign	Likely benign	
Most likely phenotype	Complex phenotype/SPG pure or complex		SPG	–	–		SPG/neuropathy	–	–	

SPG spastic paraplegia.

Key factors supporting causality include: (i) variant type (missense/truncating), (ii) localization within the motor domain, (iii) population frequency, (iv) *de novo* occurrence or segregation with disease, and (v) phenotype characteristics, including severity and age at onset. Most reported pathogenic variants are missense changes within the motor domain, typically associated with early onset and a more severe phenotype (see Supplementary material 2). In rare disorders such as KAND (mainly autosomal dominant), pathogenic variants are expected to be absent or extremely rare in population databases. For example, c.760C>T (p. R254W), identified in 20 of 177 KAND patients (14), is present in only 2 of 1,587,512 alleles in gnomAD v4.1 (in exomes only, which include data from some affected individuals).

Considering the causality in *KIF1A*, should following order (see Table 4):

- Missense or truncating variant? The missense variant is more likely pathogenic, but for truncating variants, causality can also be considered.
- Is the variant in the motor domain? The presence of missense variants in the motor domain further increases the likelihood of pathogenicity. For truncating variants, pathogenicity is not dependent on their presence in the domain.
- Does the variant have a frequency in population databases? A zero frequency further increases the likelihood of causality. In cases of extremely low frequency (corresponding to an allele count of up to 5 alleles in gnomAD v4.1), causality can still be considered. In any case, extensive segregation analysis within the family is necessary. For variants with a frequency corresponding to more than five alleles in gnomAD v4.1, segregation analysis can also be performed, but it is very likely that the variant will not segregate and is likely benign.
- Is the variant *de novo* or does it segregate with the disease? *De novo* variants and those that segregate with the disease are likely pathogenic. If the variant does not segregate with the disease but has already been reported as pathogenic and is a missense variant in the motor domain, causality remains uncertain, and the data from the literature must be carefully reviewed and considered. There may be significant intrafamilial variability in the onset of clinical signs.
- Most likely phenotype: The expected phenotype depends on the type of variant and its location in the motor domain: Missense variant in the motor domain—expected phenotype ranges from complex with early onset (most likely), to pure spastic paraplegia with early or adult onset (likely), to neuropathy/other phenotypes (very rarely). Missense variant outside the motor domain and truncating variant—most likely phenotype is pure spastic paraplegia with either early or adult onset, followed by neuropathy/other phenotypes.

## 5 Conclusion

In summary, we describe the phenotype of 18 European patients with heterozygous variants in *KIF1A*, including two variants that have not been previously documented. In light of our findings from the patient genotype–phenotype correlation, we propose a pragmatic, systematic approach to facilitate the evaluation of the causality of *KIF1A* variants. This recommendation is based on the unique characteristics of the *KIF1A* gene, which impede the application of conventional ACMG classification protocols, which are designed to assess causality. This facilitates the more effective integration of genomic data into neurological clinical practice and precision medicine.

## Data Availability

The raw data supporting the conclusions of this article will be made available by the authors, without undue reservation.

## Footnotes

1
https://www.gtexportal.org

2
http://openbioinformatics.org/annovar/

3
https://digitalinsights.qiagen.com

4
http://www.hgmd.org

5
https://www.ncbi.nlm.nih.gov/clinvar/
