# Supplementary material for: Genotype–phenotype correlations in 18 European patients with heterozygous KIF1A variants: key considerations for assessing KIF1A variant causality
Source: Front Med (Lausanne). 2026 Jan 8;12:1704209. doi: 10.3389/fmed.2025.1704209 (PMC12823886; doi:10.3389/fmed.2025.1704209)
Supplement: Supplementary file 1 [file Data_Sheet_1.docx]

**Supplementary Table 1: Pedigrees of patients/families** [wt wild type, n.t. not tested]

**Patient #1, #2, #3**


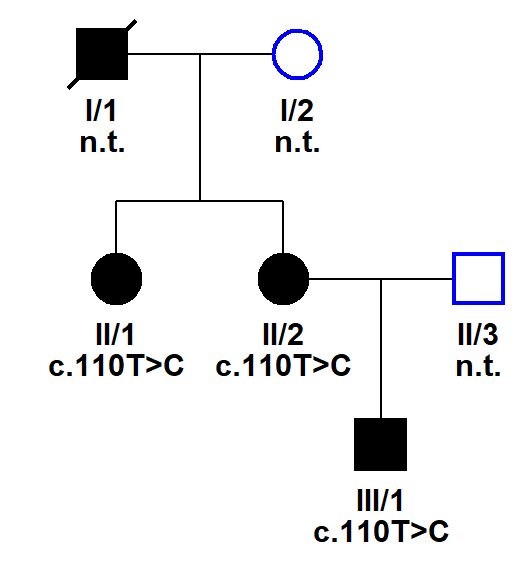


**Patient #4**


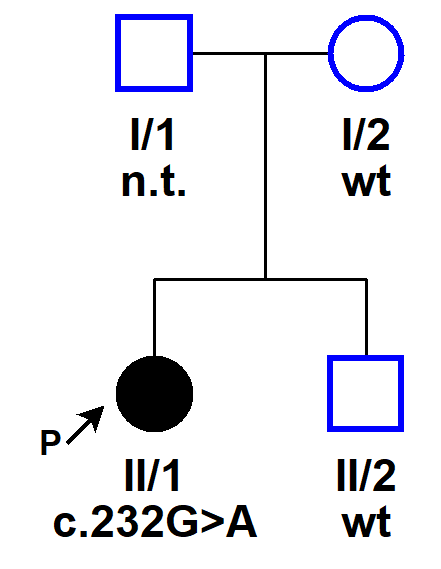


**Patient #5, #6**


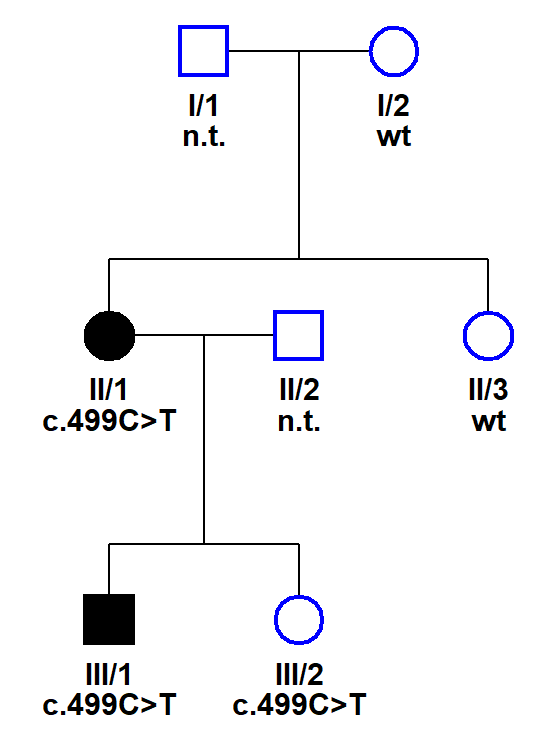


**Patient #7**


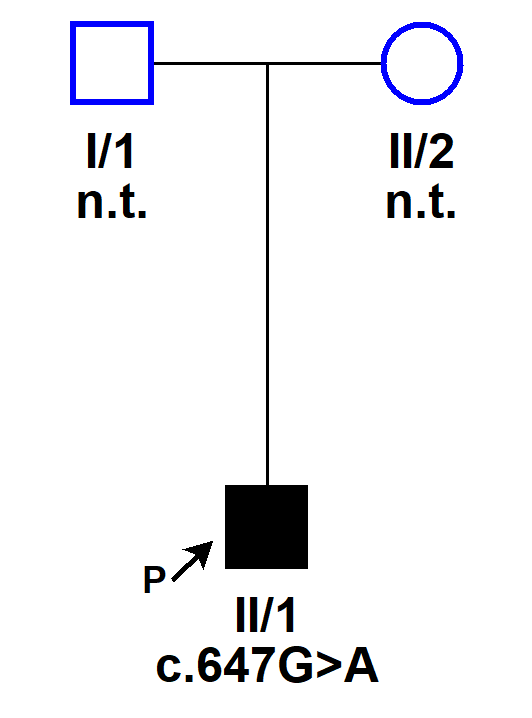


**Patient #8**


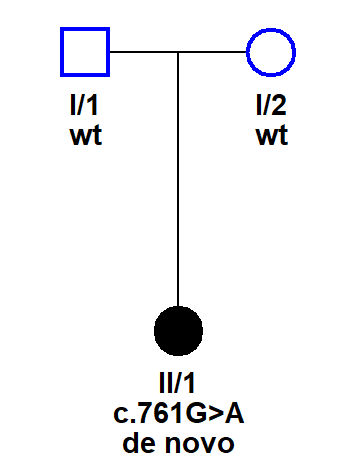


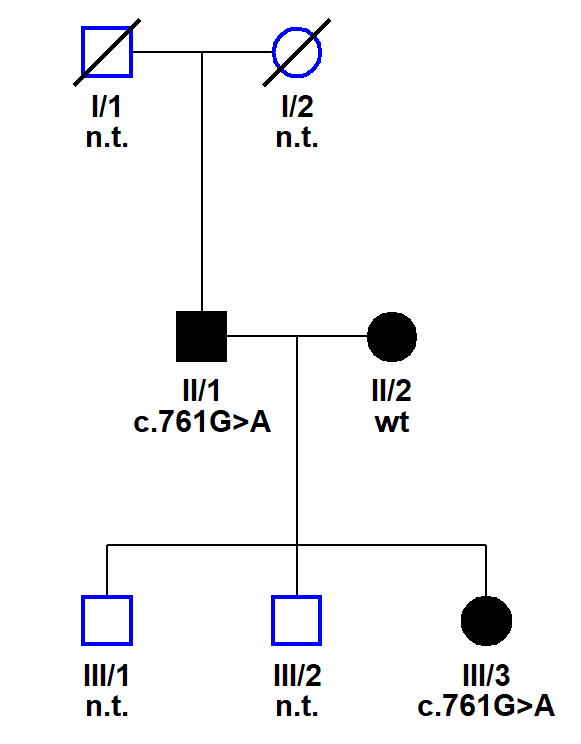
**Patient #9**

**Patient #10**


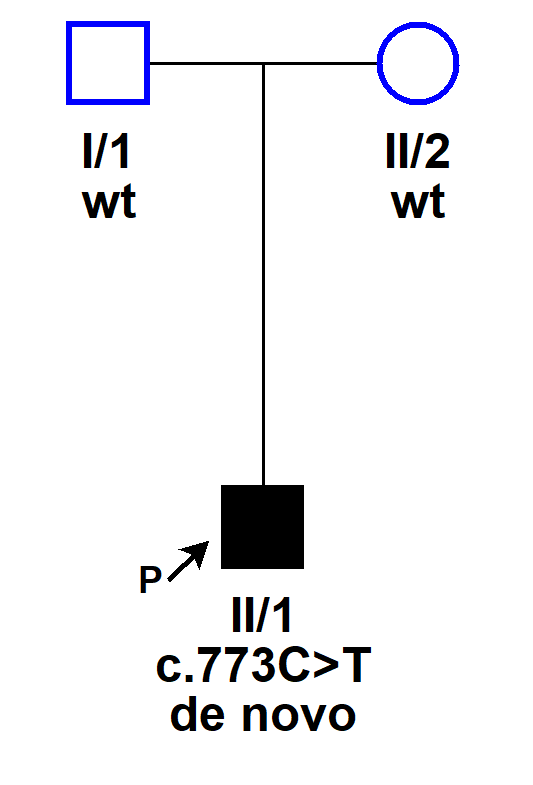


**Patient #11**


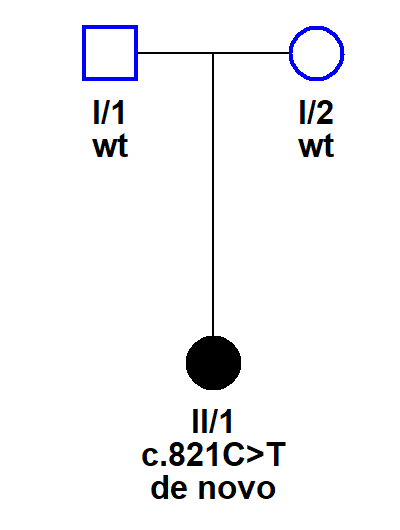


**Patient #12**


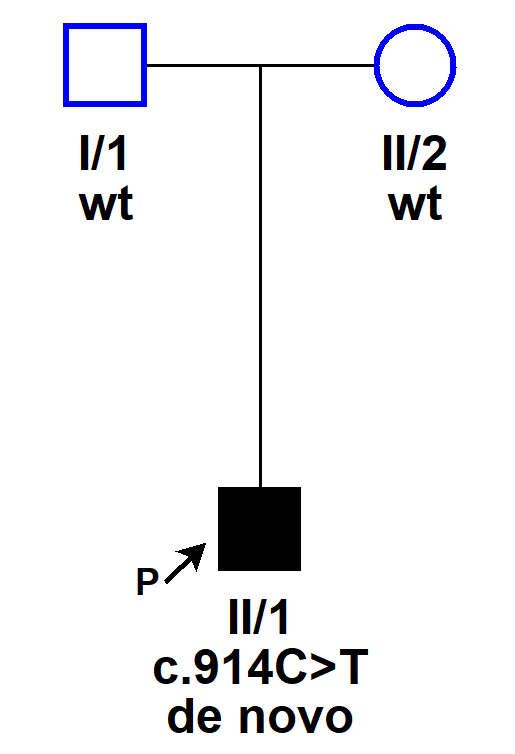


**Patient #13**


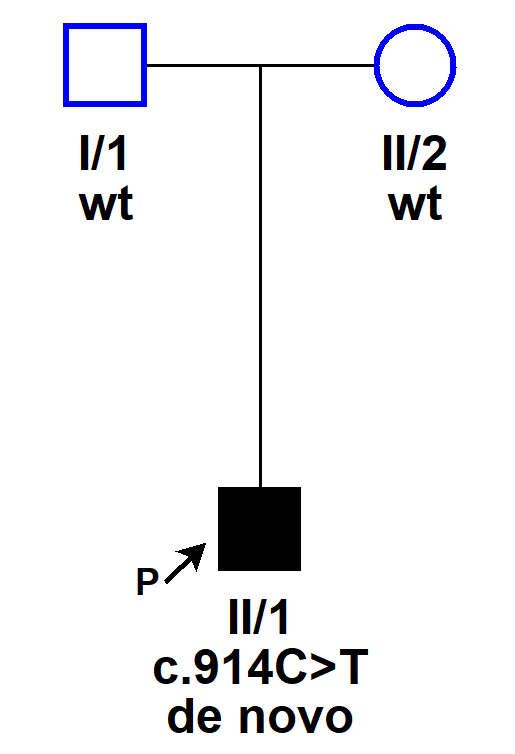


**Patient #14**


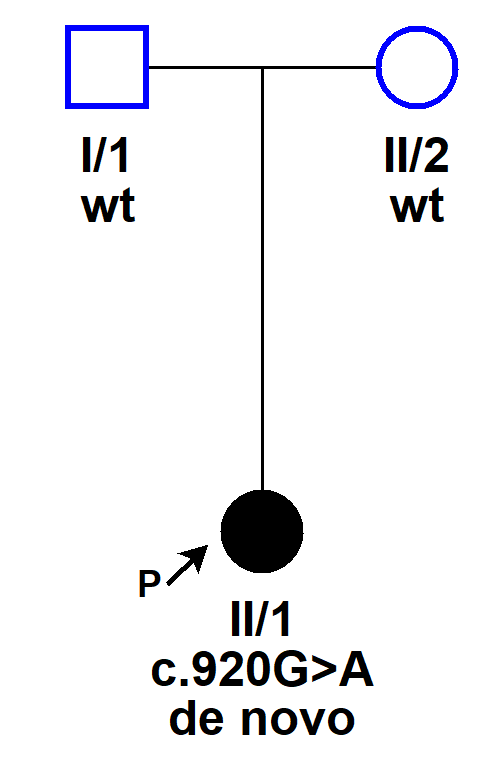


**Patient #15**


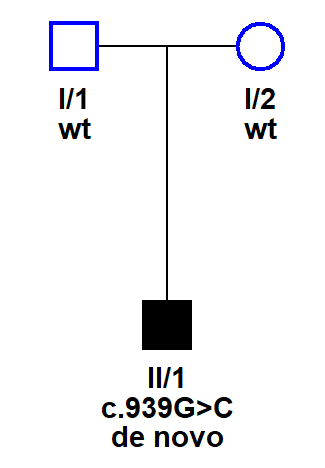


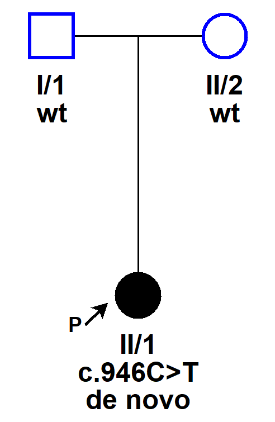
**Patient #16**

**Patient #17**


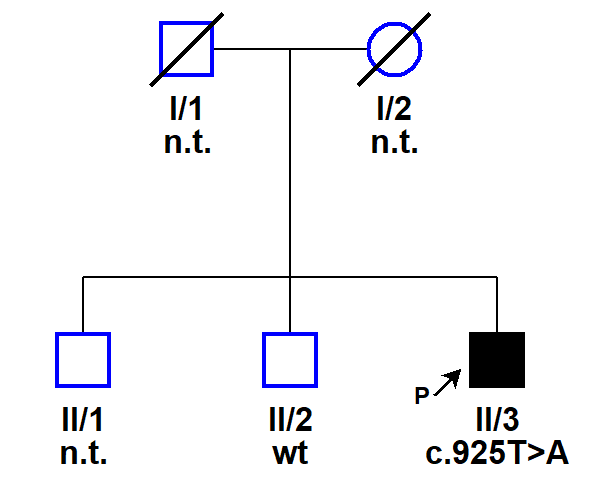


**Patient #18**

**
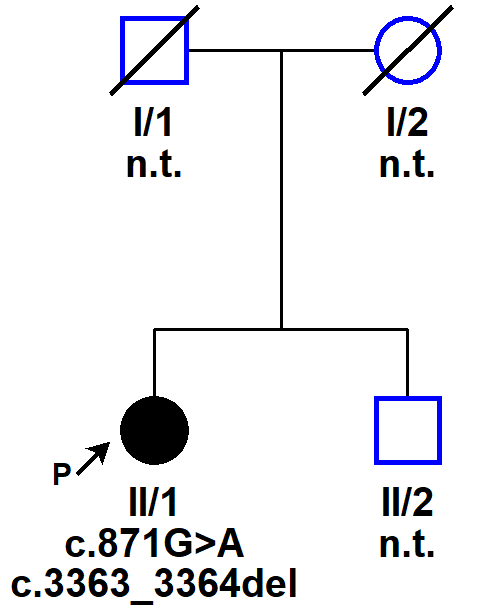
**
